# A Case Report of Primary Derealization Disorder: Diagnostic and Treatment Challenges in a Pakistani Healthcare Setting

**DOI:** 10.7759/cureus.55419

**Published:** 2024-03-03

**Authors:** Muzamil H Mirjat, Maham Kamran, Hadiqa Ashraf, Hareem Akhtar, Aasish K Lalwani

**Affiliations:** 1 Psychiatry, Jinnah Postgraduate Medical Centre, Karachi, PAK; 2 Orthopedics, Jinnah Postgraduate Medical Centre, Karachi, PAK; 3 Internal Medicine, Jinnah Postgraduate Medical Centre, Karachi, PAK; 4 Psychiatry and Behavioural Sciences, Jinnah Postgraduate Medical Centre, Karachi, PAK

**Keywords:** primary derealization, dsm 5, depersonalization disorder, child and adolescent psychiatry, adolescent

## Abstract

Depersonalization and derealization symptoms are often transient. Recurrent and persistent symptoms can result in a diagnosis of depersonalization/derealization disorder (DDD). Here, we reported a case of a 24-year-old adult male whose presentation was consistent with primary derealization disorder only. He was referred with his consent by an ophthalmologist and neurologist for psychiatric opinion for the complaints of blackish discoloration of his vision for the last two years and feeling of unreality towards his surroundings for the last one and a half years in the absence of any comorbid physical illness and mental disorder. The patient was treated with fluoxetine, Lamotrigine, and psychotherapy, but there was only some improvement reported in his distress; however, primary complaints remained unchanged.

## Introduction

Depersonalization-derealization syndrome is a relatively rare disorder with a 1-2% prevalence in epidemiologic samples. Also, patients often have difficulty narrating their symptoms, which leads to a late diagnosis of this syndrome [1,2]. As per the Diagnostic and Statistical Manual of Mental Disorders, Fifth Edition (DSM-5) and DSM‐5 Text Revision (DSM‐5‐TR) [3], depersonalization is defined as experiences of unreality, detachment, or being an outside observer concerning one’s thoughts, feelings, sensations, body, or actions (e.g., perceptual alterations, distorted sense of time, unreal or absent self, emotional and/ or physical numbing), and derealization is experiences of unreality or detachment concerning surroundings (e.g., individuals or objects are experienced as unreal, dreamlike, foggy, lifeless, or visually distorted). Transient symptoms of derealization and/or depersonalization can be reported by a variety of patients, including anxiety-spectrum disorder, depressive disorder, PTSD, use of psychoactive substances, and some physical illnesses, including head trauma and temporal lobe epilepsy [2,3]. However, persistent symptoms of exclusively derealisation are not diagnosed separately but rather included as part of depersonalization derealisation syndrome as per current diagnostic guidelines. Such presentation and symptoms pose diagnostic difficulties and management challenges for patients with a rare presentation of depersonalization-derealisation syndrome, considering the lack of established pharmacological and non-pharmacological management guidelines for such patients. These challenges even become more difficult to address in developing countries like Pakistan, where there are already limited trained care providers and available pharmacological options. Also, because of the inconsistent and complex nature of these symptoms, which are related to physical illnesses, patients undergo extensive and exhausting assessments, investigations, and healthcare visits for diagnostic and management purposes before being referred to psychiatric services.

Here, the case of a 24 years old adult male is reported who came with the complaint of gradually worsening blackish discoloration of his vision in both eyes for the last two years and feeling of estrangement towards his surroundings for the last one and a half years in the absence of any underlying physical or mental illness. There was no history of use of psychoactive substances, fever, fits, or trauma to the head. Hence, his findings were consistent with derealization disorder. He was referred by an ophthalmologist and neurologist for psychiatric opinion. The patient was then treated with fluoxetine, Lamotrigine, and psychotherapy. However, the primary complaints remained unchanged, with only some improvement in his distress.

## Case presentation

A 24-year-old male university graduate first sought medical attention from an ophthalmologist two years ago when he spontaneously and gradually developed blackish or darkish discoloration of vision. The discoloration was initially noticed on the left eye, but it worsened over the next few days to involve both eyes. The visual disturbances were exacerbated by stress and persisted irrespective of time, place, or position. There were no associated symptoms, e.g., loss of consciousness, headache, double vision, or near/farsightedness, and his visual acuity was intact. These symptoms extremely disturbed the patient, and he developed a fear of losing his vision. On examination, the ophthalmologist found no abnormalities, and his investigations, which included a slit lamp examination, color vision tests, and optical coherence tomography, were unremarkable. He was referred to a neurologist, and a detailed evaluation, including a magnetic resonance imaging (MRI) of the brain, was taken, which was unremarkable. The patient underwent relevant investigations, including complete blood count, serum urea, creatinine, electrolytes, and thyroid stimulating hormone (TSH) levels, which were reported within normal limits.

He was then referred to a psychiatrist, to whom the patient stated that, in addition to the visual symptoms, he also suddenly developed a very strange feeling of unreality towards his surroundings around 16 months ago. He started feeling that people and places around him, including those with which he was familiar, were not real. This hindered his ability to connect with his surroundings emotionally. He would pinch himself to check whether he was dreaming or not, touch cold or hot objects, and smell different fragrances to assess his surroundings. The patient denies ever experiencing feelings of unreality towards his presence or self. These symptoms were constant and persistent and not associated with any significant depressive, obsessive, psychotic symptoms or substance use, and he maintained an interest in daily activities. However, he did experience a lot of stress regarding his condition. He had occasional episodes of extreme distress regarding his condition, in which he would cry, feel the urge to scream, and pull out his hair. However, he continued his routine activities, as doing so temporarily reduced his distress. He had noticed a worsening of his complaints with any stress, especially when he got the flu three months ago.

Before the presenting complaint, around five years ago, the patient reported experiencing similar brief episodes of unreality towards his surroundings, lasting for a few hours to a day or two. However, these symptoms were not as severe or persistent and resolved spontaneously, especially after sleeping, so the patient did not seek medical help. There is no history of emotional or psychological trauma or any other significant past psychiatric illness.

There is no history of psychoactive substance use, fever, fits, or trauma to the head before or after the onset of his current condition. On mental status examination, the patient appeared oriented and well-groomed. Rapport was established and maintained throughout the interview. The speech was relevant and coherent. Cognition and insight were intact.

All other examinations were unremarkable. He was diagnosed based on DSM-V, The International Statistical Classification of Diseases and Related Health Problems, 10th revision (ICD-10), and The International Statistical Classification of Diseases and Related Health Problems, 11th revision (ICD-11), diagnostic criteria with depersonalization-derealization syndrome or disorder, exclusively derealization in this case.

The patient was referred to a psychologist, where Cognitive behavioral therapy was started, and she was also prescribed Fluoxetine 20 mg once daily (OD), after which the associative stress was improved. However, symptoms of derealisation did not change. Therefore, the fluoxetine dose was optimized to 40 mg OD after four weeks. On a follow-up visit, after one month, he reported sexual side effects with no improvement in primary symptoms. Therefore, the dose was tapered down to 20 mg, which was tolerated well and improved the distress. After more than one month of follow-up, there was good compliance to medications with no change in clinical symptomatology. Therefore, the tablet Lamotrigine was added and optimized to 50 mg OD for a month, which resulted in no change in symptoms, so it was discontinued after continuing for two months. The patient reported improvement in distress only within four weeks of the commencement of treatment; otherwise, primary complaints remained unchanged. After more than six months of treatment, frequent sessions with a psychologist, and a detailed understanding of his symptoms, as well as getting insight into the perceived and actual severity of symptoms through informational care and reading literature from various sources, he coped well with his complaints, married, and started working, though black and white vision remained a cause of distress. The primary disorder of derealisation did not improve after six months of treatment with Fluoxetine 20 mg OD and CBT, and eventually, the patient lost to follow-up. However, on his last follow-up visit, he reported significant improvement in distress, which was associated with primary complaints, and had better insight and understanding of his illness. This was reassuring and helpful for him in addressing the diagnostic challenge and preventing him from further unnecessary investigations and healthcare visits.

## Discussion

Depersonalization is characterized by a feeling of detachment from one’s own thoughts, body, emotions, actions, and sensations as though observing oneself from a distance and/or having an altered sense of time and place, while derealisation is identified as a feeling of detachment from one’s surrounding and experiencing the world as unreal, dreamlike and visually distorted [2]. 

We present a case of derealization without depersonalization, a phenomenon about which sparse epidemiology is known from past studies [4]. The prevalence rate of depersonalization-derealization disorder is approximately 2% worldwide, with a male-female ratio of 1:1, and usually peaks around 16 years of age [2]. One of the reasons for its low prevalence is that clinicians are unfamiliar with the clinical presentation of this syndrome and hence consider it secondary to other psychiatric disorders (Post-Traumatic Stress Disorder, Anxiety, or Fear-Related Disorder) [5,6]. This syndrome does not directly occur as a result of substance abuse and medications [6], and the patient in our case presents with intact reality testing in the absence of any syndromic psychiatric illness or substance use, which makes it more likely to be a case of primary derealisation syndrome.

Multiple risk factors can predispose to this syndrome, such as stress, behavioral inhibition, different types of abuse (physical, psychological, sexual, and childhood trauma), migraines, seizures, and head injury [2]. Some particular symptoms occur exclusively in derealization, such as when the patient experiences visual disturbances with surroundings, viewing them as colorless or blurry, similar to the patient in our case who presents with “blackish discoloration of vision [2,7]. Moreover, they believe everything around them is happening in a dream. These manifestations can lead to significant social, occupational, and other functional disabilities [2]. However, the patient did not experience this phenomenon in the present case.

There is very minimal literature present on derealization, such as a survey conducted among a random sample of 1008 adults in rural eastern North Carolina by telephone that included questions about experiences of depersonalization or derealization in the past year, in which 14.4% of individuals reported symptoms of derealization [8]. As of yet, there is no definitive licensed treatment for this disorder. Nevertheless, there is some proof that the use of selective serotonin reuptake inhibitor (SSRI) antidepressants, and more recently, the combination of Lamotrigine and SSRIs, has shown more beneficial results [9]. Furthermore, there was a clinical trial where 32 patients with Depersonalisation Disorder were prescribed Lamotrigine as an add-on therapy to SSRI, and results showed that fifty-six percent of patients had a Cambridge Depersonalisation scale score reduction of more than or equal to 30% [10]. Also, there has been some evidence that shows that various psychotherapies, including psychodynamic, cognitive-behavioral, hypnotherapeutic, and some other therapies, can also be used to treat this [7].

The patient in our case was seen to be in distress for a long time, undergoing rigorous investigations and yet remaining clueless about his diagnosis, leading to prolonged suffering. Therefore, when the patient was told about his diagnosis and given information about the disorder, its causes, severity, possible management strategies available, and outcomes, it was very reassuring for the patient. He was greatly relieved, which ultimately led to improvement in his condition and compliance with continued treatment, i.e., Fluoxetine 20mg OD and follow-up advice, including psychotherapy. So, to conclude, prompt diagnosis and treatment lead to a better prognosis, reduced distress, and the avoidance of unnecessary resources and financial burden on the patient.

## Conclusions

Primary derealization disorder is a relatively under-researched and under-reported clinical phenomenon. Little is known about its etiology, prevalence, predisposing factors, associated features, familial patterns, and treatment. Complex presentation and symptoms pose diagnostic difficulties and management challenges for patients, as seen in our case study with a rare presentation of derealisation syndrome, because of the lack of established management guidelines and expertise. Hence, considering its chronic fluctuating course, the severity of distress and impairment caused, and diagnostic and treatment challenges, further research is necessary to understand its etiology, course, and optimal treatment guidelines. This will help to avoid unnecessary investigations and delays in seeking mental health care, rational use of limited resources, and financial burden on the patient.
